# Treatment-adjusted prevalence to assess HIV testing programmes

**DOI:** 10.2471/BLT.21.286388

**Published:** 2021-09-30

**Authors:** Beth A Tippett Barr, David Lowrance, Cheryl Case Johnson, Rachel Clare Baggaley, John H Rogers, Shirish K Balachandra, Joseph Barker, Thokozani Kalua, Sudhir Bunga, Daniel Low-Beer, Danielle Payne, Marc G Bulterys, Andreas Jahn

**Affiliations:** a US Centers for Disease Control and Prevention, Center for Global Health, PO Box 606, Village Market, 00621 Nairobi, Kenya.; b Global HIV, Hepatitis and STI programmes, World Health Organization, Geneva, Switzerland.; c US Centers for Disease Control and Prevention, Division of Global HIV & Tuberculosis, Zimbabwe.; d Ministry of Health, Department of HIV/AIDS, Lilongwe, Malawi.; e US Centers for Disease Control and Prevention, Division of Global HIV & Tuberculosis, Juba, South Sudan.; f US Centers for Disease Control and Prevention, Division of Global HIV & Tuberculosis, Lilongwe, Malawi.; g International Training and Education Center for Health (I-TECH), University of Washington, Seattle, United States of America.

## Abstract

Scale-up of human immunodeficiency virus (HIV) testing and antiretroviral therapy (ART) for people living with HIV has been increasing in sub-Saharan Africa. As a result, areas with high HIV prevalence are finding a declining proportion of people testing positive in their national testing programmes. In eastern and southern Africa, where there are settings with adult HIV prevalence of 12% and above, the positivity from national HIV testing services has dropped to below 5%. Identifying those in need of ART is therefore becoming more costly for national HIV programmes. Annual target-setting assumes that national testing positivity rates approximate that of population prevalence. This assumption has generated an increased focus on testing approaches which achieve higher rates of HIV positivity. This trend is a departure from the provider-initiated testing and counselling strategy used early in the global HIV response. We discuss a new indicator, treatment-adjusted prevalence, that countries can use as a practical benchmark for estimating the expected adult positivity in a testing programme when accounting for both national HIV prevalence and ART coverage. The indicator is calculated by removing those people receiving ART from the numerator and denominator of HIV prevalence. Treatment-adjusted prevalence can be readily estimated from existing programme data and population estimates, and in 2019, was added to the World Health Organization guidelines for HIV testing and strategic information. Using country examples from Kenya, Malawi, South Sudan and Zimbabwe we illustrate how to apply this indicator and we discuss the potential public health implications of its use from the national to facility level.

## Résumé

Le dépistage du virus de l'immunodéficience humaine (VIH) et le traitement antirétroviral (TAR) pour les personnes vivant avec le VIH ont connu un véritable essor en Afrique subsaharienne. Par conséquent, les régions touchées par une forte prévalence du VIH détectent un pourcentage moins élevé de personnes testées positives dans leurs programmes de dépistage nationaux. En Afrique orientale et australe, là où certains endroits affichent une prévalence du VIH chez l'adulte égale ou supérieure à 12%, le taux de positivité des services de dépistage nationaux est passé sous la barre des 5%. Identifier les personnes nécessitant un TAR devient donc plus coûteux pour les programmes nationaux consacrés au VIH. Pour définir les objectifs annuels, on part du principe que les taux de positivité nationaux se rapprochent du taux de prévalence au sein de la population. Cette supposition a orienté les démarches vers des méthodes de dépistage permettant d'obtenir des taux de positivité plus élevés; une tendance qui s'écarte de la stratégie des services de dépistage et de conseil à l'initiative des prestataires, utilisée à l'aube de la lutte mondiale contre le VIH. Dans le présent document, nous nous intéressons à un nouvel indicateur, la prévalence ajustée sur le traitement. Cet indicateur peut servir de référence concrète pour les pays qui souhaitent évaluer le taux de positivité attendu chez l'adulte dans un programme de dépistage, en tenant compte de la prévalence du VIH au niveau national ainsi que de la portée du TAR. Le calcul consiste à enlever les personnes recevant un TAR du numérateur et du dénominateur de la prévalence du VIH. La prévalence ajustée sur le traitement peut aisément être déterminée en fonction des données de programme et estimations de population existantes. En 2019, elle a également été ajoutée aux lignes directrices de l'Organisation mondiale de la Santé pour l'information stratégique et le dépistage du VIH. En nous inspirant d'exemples issus du Kenya, du Malawi, du Soudan du Sud et du Zimbabwe, nous expliquons comment employer cet indicateur et abordons les potentielles implications liées à son utilisation en matière de santé publique, en partant du niveau national jusqu'aux établissements.

## Resumen

La ampliación de las pruebas de detección del virus de la inmunodeficiencia humana (VIH) y del tratamiento antirretrovírico (TAR) para las personas infectadas por el VIH ha aumentado en el África subsahariana. En consecuencia, el porcentaje de personas que dan positivo en las pruebas de detección del VIH en los programas nacionales está disminuyendo en las zonas con alta prevalencia del virus. En África meridional y oriental, donde hay entornos con una prevalencia del VIH en adultos del 12 % o superior, la tasa de positividad de los servicios nacionales de pruebas de detección del VIH ha descendido a menos del 5 %. Por lo tanto, la identificación de las personas que necesitan TAR es cada vez más costosa para los programas nacionales de VIH. El establecimiento de objetivos anuales supone que las tasas de positividad de las pruebas nacionales se aproximan a las de la prevalencia de la población. Esta suposición ha generado una mayor atención a los enfoques de las pruebas que logran tasas más altas de positividad del VIH. Esta tendencia se aleja de la estrategia del asesoramiento y las pruebas que iniciaron los proveedores y que se utilizó al principio de la respuesta mundial al VIH. Se analiza un nuevo indicador, la prevalencia ajustada según el tratamiento, que los países pueden emplear como punto de referencia práctico para estimar la tasa de positividad esperada en adultos en un programa de pruebas de detección cuando se tiene en cuenta tanto la prevalencia nacional del VIH como la cobertura del TAR. El indicador se calcula eliminando del numerador y el denominador de la prevalencia del VIH a las personas que reciben TAR. La prevalencia ajustada según el tratamiento se puede estimar con facilidad a partir de los datos de los programas existentes y de las estimaciones de población, además, en 2019, se incluyó en las directrices de la Organización Mundial de la Salud para las pruebas de detección del VIH y en la información estratégica. A través de ejemplos de países como Kenia, Malaui, Sudán meridional y Zimbabue, se demuestra cómo aplicar este indicador y se discuten las posibles implicaciones para la salud pública de su uso desde el nivel nacional hasta el de los centros.

## ملخص

لقد تزايد نطاق اختبار فيروس نقص المناعة البشرية (HIV) والعلاج المضاد للفيروسات القهقرية (ART) للأشخاص المتعايشين مع فيروس نقص المناعة البشرية في جنوب الصحراء الكبرى بأفريقيا. نتيجة لذلك، فإن المناطق التي يزيد فيها انتشار فيروس نقص المناعة البشرية لديها نسبة متناقصة من الأشخاص الذين ثبتت إصابتهم بالفيروس في برامج الاختبار الوطنية لديهم. في شرق وجنوب إفريقيا، حيث توجد بيئات يصل فيها انتشار فيروس نقص المناعة البشرية بين البالغين إلى نسبة 12% وما فوق، انخفضت الإيجابية من الخدمات الوطنية لاختبار فيروس نقص المناعة البشرية إلى أقل من 5%. ولذلك أصبح تحديد من هم بحاجة إلى العلاج المضاد للفيروسات القهقرية (ART) أكثر تكلفة بالنسبة للبرامج الوطنية لفيروس نقص المناعة البشرية. يفترض تحديد الهدف السنوي أن معدلات إيجابية الاختبار الوطنية تقارب معدلات الانتشار السكاني. أدى هذا الافتراض إلى زيادة التركيز على أساليب الاختبار التي تحقق معدلات أعلى من إيجابية فيروس نقص المناعة البشرية. هذا الاتجاه هو خروج عن استراتيجية الاختبار والاستشارة التي بدأها مقدم الخدمة، والمستخدمة مبكرًا في الاستجابة العالمية لفيروس نقص المناعة البشرية. نحن نناقش مؤشرًا جديدًا، وهو الانتشار القائم على ضبط العلاج، والذي يمكن للدول استخدامه كمعيار عملي لتقدير الإيجابية المتوقعة للبالغين في برنامج الاختبار عند التعبير عن انتشار فيروس نقص المناعة البشرية على المستوى الوطني، والتغطية المضادة للفيروسات القهقرية. يتم حساب المؤشر عن طريق حذف الأشخاص الذين يتلقون العلاج المضاد للفيروسات القهقرية من بسط ومقام انتشار فيروس نقص المناعة البشرية. يمكن تقدير الانتشار القائم على ضبط العلاج بسهولة من بيانات البرنامج الحالية وتقديرات السكان، وفي عام 2019، تمت إضافته إلى إرشادات منظمة الصحة العالمية لاختبار فيروس نقص المناعة البشرية والمعلومات الاستراتيجية. وباستخدام أمثلة من الدول من كينيا وملاوي وجنوب السودان وزيمبابوي، نوضح كيفية تطبيق هذا المؤشر، ونناقش الآثار المحتملة على الصحة العامة لاستخدامه من المستوى الوطني إلى مستوى المرفق.

## 摘要

在非洲撒哈拉沙漠以南地区,对艾滋病毒感染者进行人体免疫缺陷病毒 (HIV) 检测和抗逆转录病毒治疗 (ART) 的规模正在不断扩大。结果发现,国家检测计划实施期间,在艾滋病患病率高发的地区,检测呈阳性的人数比例正在下降。在东非和北非,成人艾滋病患病率超过 12% 的地方,国家艾滋病检测计划中的阳性率下降了 5%。对于国家艾滋病毒计划来说,确定那些需要抗逆转录病毒疗法的人的所需成本正变得越来越高。制定的年度目标通常假定国家检测计划中的阳性率接近该地区的患病率。这一假设促使人们更加重视能够提高艾滋病毒阳性率的检测方法。这一趋势背离了全球艾滋病毒应对早期使用的由提供者发起的检测和咨询策略。我们讨论一种新的指标,即调整治疗后的患病率,在考虑全国艾滋病患病率和 ART 覆盖率时,各国可将其作为评估检测计划中成人阳性率的实用基准。该指标的计算方法是将接受抗逆转录病毒治疗的人数从艾滋病毒患病率的分子和分母中去除。调整治疗后的患病率可以很容易地从现有计划数据和人口估计数中估计出来,且在 2019 年已被添加到世界卫生组织艾滋病毒检测和战略信息指南中。我们以津巴布韦、肯尼亚、马拉维和南苏丹的国家为例,说明如何应用这一指标,并讨论从国家到设施层面使用该指标的潜在公共卫生影响。

## Резюме

Расширение масштабов тестирования на вирус иммунодефицита человека (ВИЧ) и антиретровирусной терапии (АРТ) для людей, живущих с ВИЧ, увеличивается в странах Африки к югу от Сахары. В результате в регионах с высокими показателями распространения ВИЧ наблюдается снижение доли населения с положительным результатом теста в рамках национальных программ тестирования. В восточной и южной частях Африки, где распространенность ВИЧ среди взрослого населения составляет 12% и выше, положительные результаты теста на ВИЧ в рамках национальных программ тестирования опустились ниже 5%. Таким образом, выявление лиц, нуждающихся в АРТ, становится более дорогостоящим для национальных программ по борьбе с ВИЧ. Ежегодная постановка целей предполагает, что процент лиц с положительным результатом теста в рамках национальных программ тестирования приблизительно соответствует проценту распространенности среди населения в целом. Это предположение вызвало повышенное внимание к методам тестирования, которые достигают более высоких показателей распространенности ВИЧ-инфекции. Эта тенденция является отступлением от стратегии тестирования и консультирования по инициативе поставщиков услуг, которая использовалась на раннем этапе глобальных мер борьбы с ВИЧ. Авторы обсуждают новый показатель распространенности с поправкой на лечение, который страны могут использовать в качестве практического ориентира для оценки ожидаемого процента взрослого населения с положительным результатом теста в рамках программы тестирования с учетом как распространенности ВИЧ на национальном уровне, так и охвата АРТ. Показатель рассчитывается путем исключения лиц, получающих АРТ, из числителя и знаменателя доли ВИЧ-инфицированного населения. Долю ВИЧ-инфицированного населения с поправкой на лечение можно легко оценить на основе существующих данных по программе и демографических оценок, которые в 2019 году были добавлены в рекомендации Всемирной организации здравоохранения по тестированию на ВИЧ и стратегической информации. Используя примеры из Зимбабве, Кении, Малави и Южного Судана, авторы проиллюстрируют применение этого показателя, а также обсудят возможные последствия его использования для общественного здравоохранения на национальном уровне и уровне учреждения.

## Introduction

**Fig. 1:**
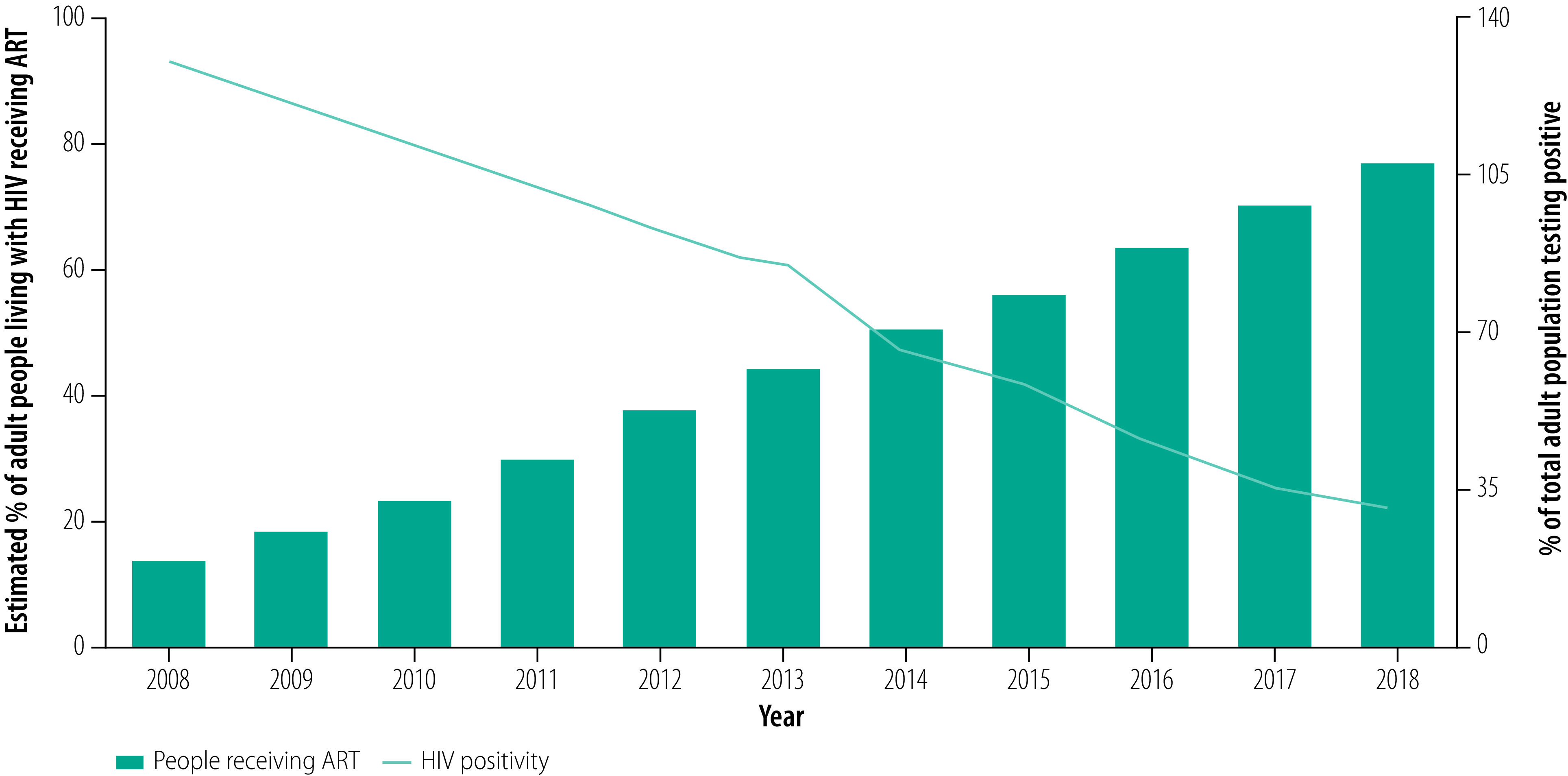
Proportion of the adult population positive for HIV infection in the national testing programme, Malawi, 2019

ART: antiretroviral therapy; HIV: human immunodeficiency virus.

Source: We obtained the data from the Malawi Ministry of Health.

Globally, there has been substantial scale-up of human immunodeficiency virus (HIV) testing services and antiretroviral therapy (ART), and it is now estimated that 78% (16 million) of the 20.6 million people living with HIV in eastern and southern Africa are receiving treatment.^1^ As a result, countries or districts with high HIV prevalence in sub-Saharan Africa are now finding a decline in positivity (that is, the proportion of people tested who are positive) in their national HIV testing programmes.^2^^–^^5^ For example, an analysis of over 13 million tests conducted primarily in health facilities in Kenya between July 2017 and June 2018 found that only 1.4% were positive.^6^ This figure compares with a national HIV prevalence in adults of 4.5% (1 390 000 people in the population of 30 888 880) in 2019.^7^ In seven out of 10 African nations with adult HIV prevalence of 10% and above, the positivity from the national HIV testing programme has been reported as 5% or below.^2^ In Malawi, for example, the proportion of people found to be HIV positive in national testing services has declined from 13.0% (170 040) of 1 304 707 people tested in 2008 to 3.1% (139 702) of 4 474 393 people in 2018, while the annual number of tests conducted has tripled (Fig. 1; A Jahn, Ministry of Health, Malawi, unpublished data, 2020). Over the same period, the estimated proportion of people living with HIV who were receiving ART increased from 14.3% (143 350 of 1 000 000 people) to 76.9% (769 179 of 1 000 000 people).^8^

This trend is encouraging, as it signals rapid progression towards the global 95–95–95 goals for reducing HIV-associated mortality and achieving and sustaining low HIV incidence.^9^ Nevertheless, as more people living with HIV are diagnosed and access treatment, finding people with undiagnosed HIV becomes progressively more difficult and expensive.^5^ Provider-initiated testing and counselling approaches were recommended by the World Health Organization (WHO) in 2007.^10^ At that time, positivity in national HIV testing programmes either reflected the prevalence in the general population, such as healthy women attending antenatal clinics, or the much higher prevalence in those attending tuberculosis or sexually transmitted disease services.

In this article we discuss the use of a new indicator, which we named treatment-adjusted prevalence. The indicator serves as a practical benchmark for the expected yield of HIV positivity in an adult testing programme when accounting for both national HIV prevalence and ART coverage. We chose the label treatment-adjusted over status awareness-adjusted as it is the aim of HIV programming to achieve virtual elimination of disease, and it is only once ART is initiated that viral load declines and onward transmission decreases.^9^ By explaining the application of this indicator with examples from sub-Saharan Africa, we hope to promote its use by national programmes and implementing organizations at subnational level.

## Challenges

The expectation that the prevalence of HIV in adults can be used to approximate HIV positivity has resulted in an increased focus on ways to increase the yield of HIV testing services.^11^^–^^17^ Such focused, high-risk, high-yield approaches are important in HIV programmes, but may not provide the volume of cost-efficient testing needed in sub-Saharan Africa to reach the majority of people with unidentified HIV infection.^5^ Declining HIV positivity, now a widespread finding in many national testing programmes, has led several countries in sub-Saharan Africa to start implementing risk-screening tools. The aim is to shift away from provider-initiated testing, to strategies which prioritize testing only for those most likely to test HIV positive.^18^^–^^22^ To date, such strategies have had variable results, with some programmes reporting that screening tools are missing too many people living with HIV who would otherwise have been tested under provider-initiated testing.

In the context of enhanced quality assurance and quality control efforts,^4^^,^^23^^–^^25^ many national HIV programmes in sub-Saharan Africa have begun reviewing the performance of their testing strategies.^26^^–^^28^ These countries have also adopted the WHO recommended three-test strategy, which requires three consecutive reactive tests to provide a positive diagnosis and enables programmes in all settings to achieve at least a 99% positive predictive value despite declining HIV positivity.^2^

With this progress in coverage of testing services, HIV programmes in sub-Saharan Africa should interpret national and subnational data and use it to guide decision-making. Countries need to consider how the rising proportions of people living with HIV who are aware of their HIV status and receiving ART will result in declining positivity and fewer undiagnosed people in need of testing services.

The challenge faced by decision-makers is how to determine appropriate benchmarks for the yield of HIV testing that can be applied to programme management and target-setting. Any indicator would need to be readily understood and applied across the national programme, including facility- and community-based services and the higher administrative levels, and including donor partner-supported programmes. As illustrated in Fig. 1, a declining yield of testing may not necessarily be an indicator of declining programme performance but may be a reflection that treatment coverage is reaching saturation. The concept of treatment-adjusted prevalence thus characterizes the remaining number of undiagnosed people living with HIV in a population. This approach could help programmes to measure their testing yield, while also considering awareness of HIV status and ART coverage among people living with HIV.

## Treatment-adjusted prevalence

Treatment-adjusted prevalence (*TAP*) is a way of removing those receiving ART from the numerator and denominator of HIV prevalence and can be readily estimated from existing programme data and population estimates, using the equation:
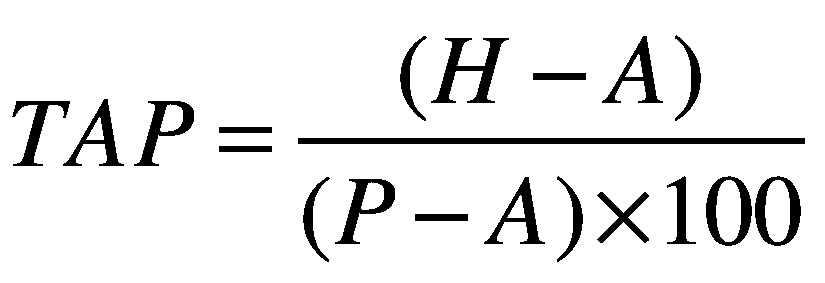
(1)where *H* is number of adults living with HIV, *A* is number of adults living with HIV and receiving ART and *P* is total adult population. The indicator has been adopted by WHO in its strategic information and guidance on HIV testing services^2^^,^^3^ and is included in its HIV testing services dashboard for 45 priority countries.^29^ Anecdotal reports, however, indicate that treatment-adjusted prevalence has not yet been widely applied at national or subnational level to guide decision-making. This lack of adaption may be due in part to the disruption of the coronavirus disease 2019 pandemic but also to the time it takes for diffusion of innovation. We compare the application of this indicator in different settings, using population data from western Kenya, Malawi, South Sudan and Zimbabwe, and we discuss the potential public health implications. We selected these four countries for this analysis to illustrate the utility of treatment-adjusted prevalence across countries with differing HIV epidemics (Box 1).

Box 1 **Profile of HIV infection in four sub-Saharan African countries**

In Kenya (adult population: 30 888 800), 1 390 000 adults (4.5%) were positive for HIV infection in 2019 according to UNAIDS estimates.^8^ The proportion of adults living with HIV who were receiving ART was 75.0% (1 042 164 people). HIV prevalence in the counties around Lake Victoria (formerly known as Nyanza province) was 12.7% (490 000 of 3 858 268 adults), making this the highest burden area in the country.^7^

Malawi (adult population: 11 235 955) also has a generalized HIV epidemic, with the health ministry reporting an estimated 1 000 000 people infected with HIV, a national prevalence of 8.9% in 2019. Prevalence was higher in the Southern region and urban areas (17.7% in Blantyre city, for example). Nationwide ART coverage was 78.5% (784 948 people).^8^

In comparison, South Sudan (adult population: 7 320 000) had an estimated national HIV prevalence of only 2.5% (183 000 people) in 2019 and ART coverage of 18.2% (33 253 people), according to data from UNAIDS.^8^

Zimbabwe (adult population: 9 921 875) has high HIV prevalence, with health ministry reports in 2019 estimating 12.8% of adults were HIV infected (1 270 000 people). Nationwide coverage of ART in adults was 79.8% (1 014 039).^8^

ART: antiretroviral therapy; HIV: human immunodeficiency virus; UNAIDS: Joint United Nations Programme on HIV/AIDS.

For example, in Zimbabwe in 2018, implementing partners reported an average testing yield of 5.1% (61 619 of 1 197 113 people) in provider-initiated testing they supported (B Makunike-Chikwinya, International Training and Education Center for Health, unpublished data, 2018). This compares with an estimated adult national prevalence of 12.8% (1 270 000 of 9 921 875 adults) in 2019.^30^ Yet, in a context where ART coverage approached 80% of all people living with HIV, determining programme performance and effectiveness in light of 6.0% testing positivity was difficult.^31^ An appropriate benchmark to compare against was needed. Using treatment-adjusted prevalence, we can determine that the yield from provider-initiated testing was double that of the remaining HIV prevalence in the adult population not receiving ART. Similarly, in the first quarter of 2019, implementing partners in Nyanza province, Kenya, reported an overall yield of 0.8% in provider-initiated testing (K De Cock, United States Centers for Disease Control and Prevention, unpublished data, 2019). The province is the highest burden area of the country, with an estimated population HIV prevalence of 12.7% (compared with Kenya’s overall 4.5% prevalence), and with an estimated 367 000 (74.9%) of all 490 000 adults living with HIV receiving ART.^7^ In these examples of high levels of both HIV testing and ART coverage, adult HIV prevalence and testing yield do not provide enough information to evaluate the overall efficiency and impact of HIV testing services.

To understand the low testing yield found in western Kenya, we applied the same logic used in Zimbabwe in 2018 to identify the treatment-adjusted prevalence for Nyanza province (Table 1). This approach uses the following data: (i) census estimate of the adult population; (ii) modelled estimate of the number of adult people living with HIV; and (iii) estimated number of adult people living with HIV receiving ART. Both the numerator and denominator could be taken from multiple sources, including programme data and population-based HIV impact assessment survey data. However, for the purpose of this demonstration, we used the Spectrum modelling software of the Joint United Nations Programme on HIV/AIDS to estimate HIV prevalence and ART coverage.^8^ Children should be excluded from both the numerator and denominator as prevalence tends to be proportionally much lower in children than adults, and only a very low proportion of HIV testing in children younger than 15 years happens outside of prevention of mother-to-child transmission programmes.

**Table 1:** Calculation of treatment-adjusted prevalence of HIV infection in the adult population aged 15–49 years in Nyanza province, western Kenya, 2019

Variable	Women	Men	All adults^a^
Total population, extrapolated^b^	2 013 423	1 862 745	3 858 268
No. of people living with HIV^c^	300 000	190 000	490 000
Population HIV prevalence, %^c^	14.9	10.2	12.7
No. of people receiving ART^c^	247 000	119 000	367 000
Treatment coverage, %^d^	82.3	62.6	74.9
Total population not receiving ART^e^	1 766 423	1 743 745	3 491 268
No. of people living with HIV not receiving ART^f^	53 000	71 000	123 000
Treatment-adjusted prevalence, %^g^	3.0	4.1	3.5

ART: antiretroviral therapy; HIV: human immunodeficiency virus; UNAIDS: Joint United Nations Programme on HIV/AIDS.

^a^ Extrapolation may result in inconsistencies.

^b^ Derived by extrapolation from UNAIDS estimates of number of adults living with HIV and prevalence of HIV.^7^^,^^8^

^c^ UNAIDS 2019 estimates.^7^^,^^8^

^d^ Calculated from number of adults receiving ART as a proportion of number of adults living with HIV.

^e^ Calculated by subtracting number of adults receiving ART from total adult population.

^f^ Calculated by subtracting number of adults receiving ART from number of adults living with HIV.

^g ^Calculated from number of adults living with HIV not receiving ART as a percentage of total adult population not receiving ART.

Notes: For this analysis we only used data from the Spectrum modelling software of UNAIDS.^7^ Kenya has moved its administration to subnational county units rather than provinces; Spectrum data still reflect provincial estimates within which specific counties can be aligned.

**Fig. 2:**
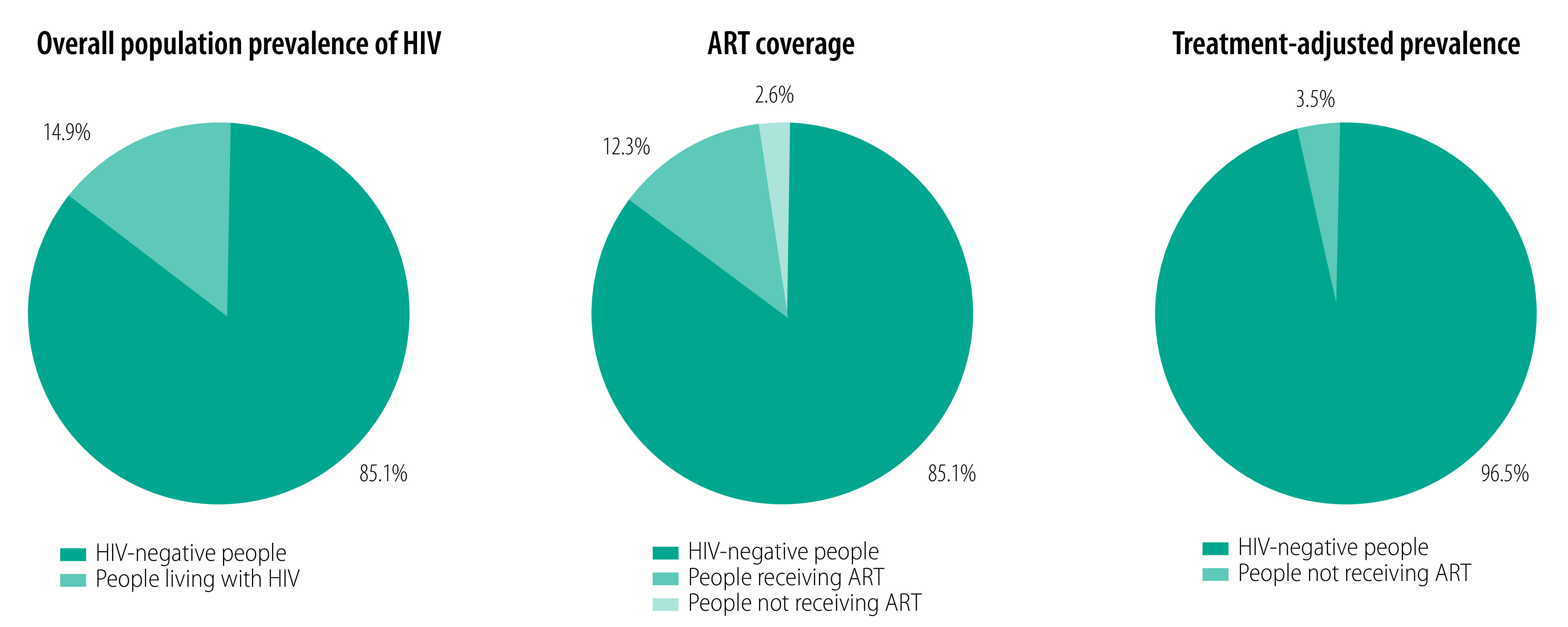
Treatment-adjusted prevalence of HIV infection in adults aged 15–49 years in Nyanza province, Kenya, 2019

ART: antiretroviral therapy; HIV: human immunodeficiency virus.

Note: See Table 1 for numerators and denominators for percentages.

Source: We obtained the data from the Kenyan Ministry of Health.

In the case of western Kenya, when the adult population who are receiving ART is removed from the numerator and denominator, the treatment-adjusted prevalence is 3.5% (3.0% in women and 4.1% in men), less than a third of the 12.7% adult prevalence estimate (Table 1).^8^ Fig. 2 illustrates how the overall prevalence of adults living with HIV is divided into those receiving ART and not receiving ART. Those not receiving ART include those who do not yet know their HIV status, those who know their status but have not started treatment, and those who were previously receiving ART but have defaulted from care. The 2.5% of the total population who are classified as people living with HIV not receiving ART becomes the 3.5% treatment-adjusted prevalence once adults receiving ART are removed from the equation.

In Table 2, we applied the same approach to the South Sudan and Zimbabwe estimates.^7^ HIV prevalence in South Sudan was 2.5%, much lower than the 12.8% documented in Zimbabwe. However, treatment coverage was higher in Zimbabwe (79.8%) than South Sudan (18.2%) and therefore, despite substantial differences in national HIV prevalence, both countries have below 3% treatment-adjusted prevalence of HIV in adults. South Sudan’s low ART coverage was similar to the coverage in Kenya, Malawi and Zimbabwe in the early 2000s, when ART coverage was just beginning to scale up. This result demonstrates that treatment-adjusted prevalence is comparable to HIV prevalence in countries with low ART coverage, but provides a marked contrast in countries with high ART coverage.

**Table 2:** Comparison of treatment-adjusted prevalence of HIV infection in the adult population aged 15–49 years in western Kenya, Malawi, South Sudan and Zimbabwe, 2019

Variable	Western Kenya	Kenya	Malawi	South Sudan	Zimbabwe
Total population, extrapolated^a^	3 858 268	30 888 800	11 235 955	7 320 000	9 921 875
No. of people living with HIV^b^	490 000	1 390 000	1 000 000	183 000	1 270 000
Population HIV prevalence, %^b^	12.7	4.5	8.9	2.5	12.8
No. of people receiving ART^b^	367 000	1 042 164	784 948	33 253	1 014 039
Treatment coverage, %^c^	74.9	75.0	78.5	18.2	79.8
Total population not receiving ART^d^	3 491 268	29 846 636	10 451 007	7 286 747	8 907 836
No. of people living with HIV not receiving ART^e^	123 000	347 836	215 052	149 747	255 961
Treatment-adjusted prevalence, %^f^	3.5	1.2	2.1	2.1	2.9

ART: antiretroviral therapy; HIV: human immunodeficiency virus; UNAIDS: Joint United Nations Programme on HIV/AIDS.

^a^ Derived by extrapolation from UNAIDS estimates of number of adults living with HIV and adult prevalence of HIV.^7^^,^^8^

^b^ UNAIDS 2019 estimates.^7^^,^^8^

^c^ Calculated from number of adults receiving ART as a proportion of number of adults living with HIV.

^d^ Calculated by subtracting number of people receiving ART from total population.

^e^ Calculated by subtracting number of people receiving ART from number of people living with HIV.

^f^ Calculated from number of people living with HIV not receiving ART as a percentage of total population not receiving ART.

## Practical application

### Selecting algorithms 

The positive predictive value of any screening test is “the probability that people with a positive result indeed do have the condition of interest.”^3^ WHO standards require all HIV tests to have at least 99% sensitivity and 98% specificity and are used in an HIV testing algorithm that achieves at least 99% positive predictive value. In practical terms, this means that there should be no more than one false positive per 100 positive diagnoses, an error which has serious consequences for both individuals and the population.^2^

To maintain such a high-quality testing service, mathematical modelling ^32^ showed that countries with adult HIV prevalence of 5% or higher could achieve a 99% positive predictive value by using two consecutive reactive tests (two-test strategy) to provide a positive diagnosis. However, for countries with adult HIV prevalence less than 5%, to achieve a 99% positive predictive value, three consecutive reactive tests (three-test strategy) were needed to provide a positive diagnosis. These WHO-recommended HIV testing strategies were first developed in 1997, when national prevalence was an acceptable indicator for determining which testing strategy a country should use. Since then, due to successful scale-up of HIV testing and ART coverage in sub-Saharan Africa, HIV epidemiology has changed, becoming more heterogeneous. Positivity in HIV testing services is well below 5% and declining in nearly all sub-Saharan Africa programmes.^2^^,^^3^ As a result, in 2019 WHO recommended all countries adopt a standard three-test strategy to ensure high-quality testing even in populations and settings within countries with HIV positivity 5% or less.^3^ Countries can use treatment-adjusted prevalence as a method to determine when to transition to the three-test strategy based on the treatment-adjusted HIV prevalence derived by removing those receiving ART from the numerator and denominator.

### Setting targets 

Using treatment-adjusted prevalence as a benchmark will allow HIV programmes to assess their effectiveness in targeted testing. The information can be readily used to analyse the quality and precision of targeted HIV testing services in each country and to improve the efficiency and effectiveness of testing approaches. For example, in Zimbabwe, the positivity observed in routine clinical testing was more than twice that of the treatment-adjusted prevalence, despite being less than half the overall adult HIV prevalence. This finding indicated that provider-initiated testing and counselling was still a high-yield, cost-efficient testing strategy. Treatment-adjusted prevalence facilitated feasible target-setting for testing programmes both in terms of the volume of tests to be conducted and a more accurate estimate of the minimum number of HIV-positive people who would be identified and subsequently linked to care. This outcome meant that the country was also able to estimate treatment costs for the following year more accurately.

Additionally, the indicator can assist in prioritizing resources to those subnational levels (districts, counties or states) which have variations in both HIV prevalence and in treatment coverage. Treatment-adjusted prevalence essentially controls for those variations at the subnational level in the same way as shown at the national level (Table 2).

### Identifying shortfalls

In subnational areas and even health facility catchment areas where the observed testing yield is below the estimated treatment-adjusted prevalence, testing programmes could be reviewed to see if more effective approaches can be implemented. As described above, in the western Kenya counties that formerly made up Nyanza province, positivity in routine HIV testing in clinical settings (0.8%) was less than a third of the treatment-adjusted HIV prevalence (3.5%). These data indicate a need for further investigation into how well routine testing practices are aligned with national and global guidance.^2^ Our rapid assessment (B Tippett Barr, Center for Global Health, Kenya, unpublished data, 2019) revealed that provider-initiated testing was not routinely offered at all service delivery points as per global guidance. Although available at patient registration points, patient testing required as much initiative on the part of the patient as the provider. However, in antenatal settings in the same facilities where provider-initiated testing was routinely implemented according to global guidance, HIV test positivity exceeded the treatment-adjusted prevalence in antenatal care. These findings further reinforce the concept of treatment-adjusted prevalence as a lower bound for effective testing strategies. If the women attending antenatal care are mostly healthy and represent a cross-section of society, logically their HIV prevalence should be lower than those who are attending a health facility with an illness. The conclusion we drew was that inconsistent offers of HIV testing in non-antenatal care settings in health facilities in western Kenya was not a cost-effective or productive strategy. One should note that low test positivity in non-antenatal care settings does not negate the need for provider-initiated testing; it may instead indicate that such testing is not being correctly implemented. As in Zimbabwe,^31^ when we conducted a careful review of site-level implementation and closed gaps in consistency and service delivery points, test positivity increased markedly. These experiences from Kenya and Zimbabwe illustrate how treatment-adjusted prevalence can be a practical benchmark for the lower bound of expected HIV positivity in any testing setting, particularly during an era of increasingly targeted testing to improve case identification.

## Limitations

As described above, a limitation of the new indicator is that it does not account for the proportion of individuals who already knew their HIV status and chose not to disclose that information during HIV testing, therefore artificially inflating the treatment-adjusted prevalence. Studies have reported that 13–68% of patients known to be positive seek re-testing before starting ART.^33^^–^^36^ Anecdotal reports also reveal that individuals already receiving ART occasionally re-test for personal reasons. However, based on field experience across multiple countries, we do not believe the proportions of people re-testing after starting ART exceeds the proportion re-testing before ART. The limitation of including people known to be HIV positive but seeking repeat testing does then not detract from the usefulness of treatment-adjusted prevalence as a lower bound for the expected yield of testing. An additional limitation to this indicator is that it excludes individuals younger than 15 years. Other approaches are needed to improve the targeting and performance of HIV testing programmes for children. National or subnational treatment-adjusted prevalence estimates are also not applicable to key populations (such as men who have sex with men or female sex workers), as these population subgroups have consistently higher HIV prevalence and often lower ART coverage than the general adult population.

We developed treatment-adjusted prevalence primarily to address questions emerging in sub-Saharan Africa; its utility has not yet been demonstrated for priority subpopulations or for other settings. The logic applied in this indicator is transferable to other settings and populations but, as with all estimates, the validity of the point estimate would depend on the accuracy of the estimates used. In all settings and populations, when treatment-adjusted prevalence is applied at subnational levels, and by default applied to smaller numbers, there will be increasing uncertainty around the point estimate produced.

## Conclusion

Treatment-adjusted HIV prevalence is a practical and simple indicator constructed from readily available data, which could guide the selection of national HIV testing algorithms and hence improve programme management and monitoring. This indicator, adopted by WHO, provides a lower bound for expected HIV testing yield in settings where coverage of ART is high. The adjustment may result in more appropriate HIV testing services and treatment targets and may help evaluate performance in heterogeneous populations. The indicator helps focus HIV testing programmes on people with undiagnosed HIV and on individuals known to be positive but who are not receiving ART. Nevertheless, treatment-adjusted prevalence should not detract from the additional focus on testing approaches for subpopulations with higher HIV risk.

Depending on the quality of data available in a country, treatment-adjusted prevalence could also be disaggregated at subnational levels, by sex or by age group. Furthermore, the indicator may be useful for monitoring the global HIV response and for prioritizing geographical regions, as it can be routinely derived when countries conduct their annual HIV modelling estimates. The development and practical application of indicators such as treatment-adjusted prevalence will become increasingly important as HIV treatment coverage approaches 100%.
